# Impact of the COVID-19 pandemic on student’ sleep patterns, sexual activity, screen use, and food intake: A global survey

**DOI:** 10.1371/journal.pone.0262617

**Published:** 2022-01-28

**Authors:** Passent Ellakany, Roberto Ariel Abeldaño Zuñiga, Maha El Tantawi, Brandon Brown, Nourhan M. Aly, Oliver Ezechi, Benjamin Uzochukwu, Giuliana Florencia Abeldaño, Eshrat Ara, Martin Amogre Ayanore, Balgis Gaffar, Nuraldeen Maher Al-Khanati, Anthonia Omotola Ishabiyi, Mohammed Jafer, Abeedha Tu-Allah Khan, Zumama Khalid, Folake Barakat Lawal, Joanne Lusher, Ntombifuthi P. Nzimande, Bamidele Emmanuel Osamika, Mir Faeq Ali Quadri, Mark Roque, Anas Shamala, Ala’a B. Al-Tammemi, Muhammad Abrar Yousaf, Jorma I. Virtanen, Annie Lu Nguyen, Morenike Oluwatoyin Folayan

**Affiliations:** 1 Mental Health and Wellness Study Group, Oaxaca, Mexico; 2 Department of Substitutive Dental Sciences, College of Dentistry, Imam Abdulrahman Bin Faisal University, Dammam, Saudi Arabia; 3 Postgraduate Department, University of Sierra Sur, Oaxaca, Mexico; 4 Faculty of Dentistry, Alexandria University, Alexandria, Egypt; 5 Department of Clinical Sciences, Nigerian Institute of Medical Research, Lagos, Nigeria; 6 Department of Social Medicine, Population and Public Health, University of California, Riverside School of Medicine, Riverside, California, United States of America; 7 University of Nigeria Nsukka, (UNN) Enugu Campus, Nsukka, Nigeria; 8 School of Medicine, University of Sierra Sur, Oaxaca, Mexico; 9 Government College for Women, Srinagar, Kashmir (J&K), India; 10 Department of Health Policy Planning and Management, University of Health and Allied Sciences, Ho, Ghana; 11 Department of Preventive Dental Sciences, College of Dentistry, Imam Abdulrahman Bin Faisal University, Dammam, Saudi Arabia; 12 Department of Oral and Maxillofacial Surgery, Faculty of Dentistry, Syrian Private University, Damascus, Syria; 13 Centre for Rural Health, School of Nursing and Public Health, University of KwaZulu-Natal, Durban, South Africa; 14 Department of Health Promotion, Faculty of Health, Medicine and Life Sciences, Maastricht University, Maastricht, The Netherlands; 15 School of Biological Sciences, University of the Punjab, Lahore, Pakistan; 16 Department of Periodontology and Community Dentistry, University of Ibadan and University College Hospital, Ibadan, Nigeria; 17 Regent’s University London, London, United Kingdom; 18 Department of Economic and Human Geography, University of Szeged, Szeged, Hungary; 19 Department of Child Oral Health, University of Ibadan, Ibadan, Nigeria; 20 Department of Preventive Dental Sciences, Dental Public Health, College of Dentistry, Jazan University, Jizan, Saudi Arabia; 21 Maternity and Childhood Nursing Department, College of Nursing, Taibah University, Madinah, Kingdom of Saudi Arabia; 22 Department of Preventive and Biomedical Science, Faculty of Dentistry, University of Science and Technology, Sanaa, Yemen; 23 Doctoral School of Health Sciences, University of Debrecen, Debrecen, Hungary; 24 Institute of Zoology, University of the Punjab, Lahore, Pakistan; 25 Faculty of Medicine, University of Turku, Turku, Finland; 26 Department of Family Medicine, Keck School of Medicine, University of Southern California, Los Angeles, California, United States of America; 27 Department of Child Dental Health, Obafemi Awolowo University, Ile-Ife, Nigeria; National Institute of Research in Tribal Health, INDIA

## Abstract

**Background:**

The education sector experienced substantial impacts during the COVID-19 pandemic resulting from worldwide restrictions.

**Purpose:**

To examine differences in the sleep patterns, sexual activity, screen use, and food intake of students and non-students during the COVID-19 pandemic.

**Methods:**

This was a global cross-sectional study conducted in the second half of 2020 using multiple social media platforms to recruit study participants globally. A close-ended questionnaire was administered anonymously in English, French, Spanish, Portuguese, and Arabic to adults ages 18 and older. The outcome variables considered in analyses were changes in sleep pattern, sexual activity, screen use, and food intake. The explanatory variable was student status categorized as students vs. non-student. T-test, chi-square, and Mann Whitney U tests were used to assess differences between student and non-student populations. One logistic regression model was built for each outcome variable. Country of residence and country income level were included in the adjusted models.

**Results:**

There were 17,008 participants of which 3,793 (22.3%) were students. Of the total sample, 4,889 (28.7%) reported changes in sleep, 4,642 (31.8%) reported increases in sexual activity, 10,278 (70.7%) reported increases in screen use, and 5,662 (40.2%) reported increases in food intake during the pandemic. Compared to non-students, students had significantly higher odds of reporting changes in sleep (AOR = 1.52), increases in sexual activity (AOR = 1.79), and increases in screen use (AOR = 1.36) but lower odds of reporting increase in food intake (AOR = 0.87).

**Conclusion:**

Students displayed higher risk of experiencing changes in sleep, sexual behavior, and screen use during the COVID-19 pandemic. This has the potential to lead to broader adverse effects on students’ overall wellbeing. The findings and implications raise further obligations on the education sector to put extra-curricular support systems in place that address COVID-19 related behavior changes that have the potential to adversely impact students’ wellbeing.

## Introduction

The academic sector has been highly impacted by the lockdowns instituted as a containment measure during the COVID-19 pandemic [1]. Lockdown patterns across the world continue to vary from total lockdown to localized lockdown, dynamic lockdown, and no lockdown [2]. Educational activities, particularly in countries that enforced total lockdown, shifted from physical presence to virtual learning using digital communication platforms to maintain social distancing, minimize in-person contact, and contain the spread of the pandemic [3–5]. Early in the pandemic, there were initial concerns expressed by students regarding difficulty accessing classes leading to the postponement of graduation or inability to complete program courses according to schedule [6]. This has the potential to strain affected student’s finances, psychological welfare, and attainment of future goals [1]. The use of digital platforms enabled students to continue to attend classes in a virtual capacity and reduce the fear of graduation delay and concerns about the withdrawal of funding before the end of the school program [3, 6]. However, distance learning has led to an uptick of behaviors among students [7] like increases in tobacco and alcohol consumption [8], changes in sexual behaviors [9, 10], or early marriages to reduce financial burdens for families in many developing countries [7, 11].

Student wellbeing during the pandemic remains an ongoing and major concern for higher education institutions across the globe [12]. The world has witnessed protests with students marching through cities and occupying buildings to express their anger and frustration over a perceived lack of mental health support received from universities [13]. There is also a surge of media reports on the rising rate of poor mental health among students as a direct result of the COVID-19 pandemic [14], with warnings from student representatives of the worsening of an existing mental health crisis among students internationally [15]. Some students are facing consequences like boredom, stress, anxiety, and depression [16]. The lack of physical contact, outlets for socializing and communication with peers and teachers, restrictions on travel, and reduced physical activities have contributed to these emotional issues [17]. Other sources of stress for some students include the inability to afford educational resources such as computers, separate rooms conducive for learning, high-speed internet for each family member who require educational facilities, and new expenses associated with the procurement of computing devices as a consequence of the change in educational modality [16, 18, 19]. Distance learning is also limited in the ability to facilitate all the learning outcomes of professional education that require hands-on practice such as medicine, dentistry, nursing, nutrition, and other allied health courses [1]. In the case of countries with partial or no lockdown, students need to maintain physical distancing and grapple with the constant fear of contracting infection or spreading it to a family member, fear of job loss, and the inability to pay for the basic life needs such as food or housing expenses [17].

These pandemic-related concerns greatly impact the routines related to daily life of students like their sleep pattern, sexual activity, screen time, and food intake [3, 8, 20]. Eating habits may change because of the impact of the pandemic on their mental/psychological health; students may have changed their eating patterns and physical activity [21, 22]. Staying at home for a long time may also result in high consumption of junk food, snacks, chocolate, and alcohol drink as well [8, 23, 24]. Also, distress and irritability resulting from the lockdown can lead to change in sleeping hours, disturbed sleep due to the overstraining, and/or nightmares [25]. Furthermore, changes in sexual desire and frequency of intercourse during the COVID-19 pandemic have been documented, while the quality of sexual life has significantly decreased [25, 26].

Though there is some evidence on how the COVID-19 pandemic has affected the behavior of students, there is little known about how behaviors differ between students and non-students. Thus, the aim of the current study was to examine differences in the sleep patterns, sexual activity, screen use, and food intake of students and non-students during the COVID-19 pandemic. We hypothesize that the pandemic had a greater effect on sleep patterns, sexual behavior, screen time, and food intake for students when compared to non-students. Understanding the ways in which stressful life conditions and public health emergencies have differential impact on students’ lives may help in tailoring appropriate and early intervention for students. Also, the information might be valuable for policy makers when developing preparedness and strategic plans during future pandemics.

## Methods

A global cross-sectional study recruited respondents through multiple social media platforms using an online survey tool from the 29^th^ of June 2020 to the 31^st^ of December 2020. The survey was distributed across several social media platforms such as Facebook, Twitter, Instagram in addition to WhatsApp groups and emails. Participants were further asked to share the links with their own networks using snowball sampling. Adults aged 18 and above were eligible to participate in the study and respondents came from 136 countries. This study was approved by the human research ethical committee of the Institute of Public Health of the Obafemi Awolowo University Ile-Ife, Nigeria (IPHOAU/12/1557). Additional ethical approvals were attained from India (D-1791-uz and D-1790-uz), Saudi Arabia (CODJU-2006F), and Brazil (CAAE N° 38423820.2.0000.0010).

The instrument used for the survey underwent content validation. The overall content validity index of the survey was 0.83. The responses collected for content validation were excluded from the final analysis. The survey instrument was administered in English, French, Spanish, Portuguese, and Arabic. The French, Spanish, Portuguese, and Arabic versions were translated and back translated from the English version. The survey was preceded by an introduction about the study team, study objectives and time needed to complete the questionnaire. This was followed by a consent form assuring participants of the confidentiality of their responses and emphasizing that their participation was voluntary and anonymous. Only participants who consented could proceed to the survey.

### Variables and data analyses

Sociodemographic data was collected and age, sex at birth (male, female, intersex), and country of residence were included in the current analyses as covariates. The outcome variables selected for this study were changes in sleep pattern (sleeping more, less, or no changes), sexual activity (more, less, or no changes), screen use (increase, decrease, no change), and food consumption (increase, decrease, no change). The outcome variables were dichotomized into changes (sleeping more, less) and no changes in sleep pattern; increase (more) and no increase (less, or no changes) in sexual activity; increase (increase) and no increase (decrease, no change) use of screen; and increase (increase) and no increase (decrease, no change) in food consumption for the logistic regression analysis. The explanatory variable was student status. Participants were asked to define their present work status and participants who selected “undergraduate student” or “post-graduate student” as a response were categorized as students and all others were categorized as non-students.

SPSS software version 23.0 (IBM Corp., Armonk, N.Y., USA) was used for statistical analysis. Descriptive statistics were calculated as means and standard deviations or frequencies and percentages. Country income level was classified according to the World Bank Data into low-income countries (LICs) with a gross national income (GNI) per capita ≤1,035 USD in 2019, lower middle-income countries (LMICs) with GNI between 1,036 and 4,045 USD, upper middle-income countries (UMICs) with GNI between 4,046 and 12,535 USD and high-income countries (HICs) with GNI≥ 12,536 USD [27, 28]. S1 Table provides a breakdown of the study countries by income level. T-test, chi-square and Mann Whitney U tests were used to assess differences in the variables between students and non-students’ populations.

Four logistic regression models were built: one for each outcome variable (change in sleep pattern, increase in sexual activity, increase in screen use, and increase in food consumption). All the explanatory variables and covariates were included in the models. Country of residence was set as random effect variable and country income level was introduced as fixed effect factor with the other study variables. The potentials for collinearity were checked and none was found. Adjusted odds ratio (AOR) and 95% confidence intervals (CI) were calculated. Significance was set at 5%.

## Results

Table 1 shows the demographic profile of the study participants. The total sample included 17,008 participants, 3793 (22.3%) of whom were students. Females made up 61.7% of the sample and the mean age of was 31.0 years (SD = 8.9). In terms of the outcomes, 4,889 people (28.7%) reported change in sleep patterns, 4,642 (31.8%) had an increase in sexual activity, 10,278 (70.7%) reported an increase in the use of screens, and 5,662 (40.2%) reported an increase in food intake.

**Table 1:** Differences between students and non-students by their sociodemographic factors and change in personal behaviors during the pandemic (N = 17,008).

Factors		Non-students N = 13,215 n (%)	Students N = 3,793 n (%)	P value	All N = 17,088 n (%)
**Sociodemographic factors**					
**Age**	**Mean (SD)**	38.8 (12.4)	23.2 (5.3)	<0.001*	31.0 (8.9)
**Sex at birth**	**Male**	5291 (40.0)	1075 (28.3)	<0.001*	6366 (37.4)
	**Female**	7825 (59.2)	2675 (70.5)		10500 (61.7)
	**Intersex**	12 (0.1)	2 (0.1)		14 (0.1)
	**No answer**	87 (0.7)	41 (1.1)		128 (0.8)
**Country income classification**	**LICs**	355 (2.7)	52 (1.4)	<0.001*	407 (2.4)
	**LMICs**	6912 (52.3)	2094 (55.2)		9006 (53.0)
	**UMICs**	2697 (20.4)	779 (20.5)		3476 (20.4)
	**HICs**	3251 (24.6)	868 (22.9)		4119 (24.2)
**Change in personal behaviors during COVID-19**					
**Change in sleep pattern**	**No changes from usual**	9711 (73.5)	2408 (63.5)	<0.001*	12119 (71.3)
	**Yes (sleeping more or less)**	3504 (26.5)	1385 (36.5)		4889 (28.7)
**Change in sexual activity**	**Decrease or no change**	8247 (73.0)	1689 (51.6)	<0.001*	9936 (68.2)
	**Increase**	3058 (27.0)	1584 (48.4)		4642 (31.8)
**Change in screen use**	**Decrease or no change**	3552 (31.5)	710 (21.7)	<0.001*	4262 (29.3)
	**Increase**	7709 (68.5)	2569 (78.3)		10278 (70.7)
**Change in food intake**	**Decrease or no change**	6617 (60.7)	1813 (56.9)	<0.001*	8430 (59.8)
	**Increase**	4290 (39.3)	1372 (43.1)		5662 (40.2)

*: statistically significant at P< 0.05.

Compared to non-students, students were younger (p<0.001), more likely to be female (p<0.001) and less likely to be from LICs (p<0.001). More students reported a change in sleep pattern (p<0.001), increases in sexual activity (p<0.001), increases in screen use (p<0.001), and increases in food intake (p<0.001).

Table 2 reports on the sex, age and income level of country of residence adjusted logistic regression models determining factors associated with change in personal behaviors reported by students during the pandemic. The results show that, compared to non-students, students had significantly higher odds of reporting changes in sleep (AOR = 1.52; 95% CI: 1.39, 1.67), increases in sexual activity (AOR = 1.79; 95% CI: 1.62, 1.97) and increases in screen use (AOR = 1.36; 95% CI: 1.23, 1.52), but lower odds of reporting increases in food intake (AOR = 0.87; 95% CI: 0.79, 0.95).

**Table 2:** Change in personal behaviors reported by students during the pandemic controlling for sex, age and income level of country of residence (N = 13,116).

Factors		Change in sleep pattern		Increase in sexual activity		Increase in screen use		Increase in food intake	
		AOR (95% CI)	P value	AOR (95% CI)	P value	AOR (95% CI)	P value	AOR (95% CI)	P value
Age		1.00 (1.00, 1.00)	0.26	0.98 (0.975, 0.982)	<0.001*	0.99 (0.98, 0.99)	<0.001*	0.983 (0.980, 0.986)	<0.001*
Sex at birth	Female	1.36 (1.27, 1.47)	<0.001*	1.22 (1.13, 1.32)	<0.001*	1.07 (0.99, 1.15)	0.08	1.26 (1.18, 1.36)	<0.001*
	Non-female	1.00	-	1.00	-	1.00	-	1.00	-
Country income classification	LIC	0.74 (0.58, 0.94)	0.01*	1.43 (1.11, 1.85)	0.005*	0.92 (0.72, 1.17)	0.47	0.70 (0.55, 0.90)	0.005*
	LMIC	0.67 (0.61, 0.72)	<0.001*	1.82 (1.66, 2.00)	<0.001*	0.85 (0.78, 0.92)	<0.001*	0.91 (0.83, 0.98)	0.02*
	UMIC	1.26 (1.15, 1.39)	<0.001*	1.17 (1.05, 1.31)	0.007*	1.37 (1.23, 1.53)	<0.001*	1.16 (1.05, 1.28)	0.004*
	HIC	1.00	-	1.00	-	1.00	-	1.00	-
Student	Yes	1.52 (1.39, 1.67)	<0.001*	1.79 (1.62, 1.97)	<0.001*	1.36 (1.23, 1.52)	<0.001*	0.87 (0.79, 0.95)	0.003*
	No	1.00	-	1.00	-	1.00	-	1.00	-

AOR: adjusted odds ratio, CI: confidence interval,

*: statistically significant at p< 0.05.

Additionally, older respondents had significantly lower odds of reporting increases in sexual activity (AOR = 0.98; 95% CI: 0.975, 0.982), increases in screen use (AOR = 0.99; 95% CI: 0.98, 0.99), and increases in food intake (AOR = 0.983; 95% CI: 0.980, 0.986). Females had significantly higher odds of reporting changes in sleep (AOR = 1.36; 95% CI: 1.27, 1.47), increases in sexual activity (AOR = 1.22; 95% CI: 1.13, 1.32), and increases in food intake (AOR = 1.26; 95% CI: 1.18, 1.36).

Participants from LICs and LMICs had lower odds of reporting changes in sleep (AOR = 0.74; 95% CI: 0.58, 0.94 and AOR = 0.67; 95% CI: 0.61, 0.72), increases in screen use (AOR = 0.92; 95% CI: 0.72, 1.17; and AOR = 0.85; 95% CI: 0.78, 0.92) and increases in food intake (AOR = 0.70; 95% CI: 0.55, 0.90 and AOR = 0.91; 95% CI: 0.83, 0.98) when compared to participants from HICs. However, participants from UMICs, had significantly higher odds of reporting changes in sleep (AOR = 1.26; 95% 1.15, 1.39), increases in sexual activity (AOR: 1.17; 95% CI: 1.05, 1.31), increases in screen use (AOR = 1.37; 95% CI: 1.23, 1.53), and increases in food intake (AOR = 1.16; 95% CI: 1.05, 1.28) when compared to participants from HICs. Participants from LICs (AOR = 1.43; 95% 1.11, 1.85), LMICs (AOR = 1.82; 95% CI: 1.66, 2.00), and UMICs (AOR = 1.17; 95% 1.05, 1.31 had higher odds of reporting an increase in sexual activity when compared to HICs during the pandemic.

## Discussion

Overall, the present study identified some significant differences in sleep patterns, sexual behaviour, screen use, and food intake between students and non-students during the COVID-19 pandemic. Students had higher odds of reporting changes in sleep, increases in sexual activity, and increases in screen use, but lower odds of reporting increases in food intake during the pandemic. The hypotheses for the study were partially confirmed.

One of the main strengths of this study was the large sample size and the global representation of the study participants as well as the diversity of the economic regions from which data were collected. We also highlighted a comparison between students and non-students, thereby being one of the few studies that provide primary data on the different ways that the pandemic has affected different subgroups within diverse populations. The ratio of students to non-students among the study participants reflects the predominance of non-students in various communities in reality. The findings could also assist institutions of higher learning, especially those in UMICs, to identify how to mitigate the impact of the COVID-19 pandemic on students’ mental health and wellness.

The study, however, has some limitations. This was a cross sectional study limiting the ability to conclude on the causal relationship between the variables we found to be association in this study. Further, the measures were self-reported with possible increase in social desirability bias. Variables such as ethnicity or race as well as religion were not investigated. Despite these limitations, the study provides new insight about the impact of the COVID-19 pandemic on students and non-students.

The observed changes in sleep pattern and increase in screen use by students have been previously reported [29, 30]. A significant deterioration in sleep quality of students during the pandemic may be due to late night browsing on social media, chatting, and checking online news from mobile devices [31] as well as the increased use of screens for educational purposes, which is also associated with late night use of electronic devices [20]. The significant increase in exposure to screens may lead to longer waking hours and reduce sleep duration as a result of blue light emitted from mobile screens, inhibiting melatonin production [32, 33]. This adjusts the sleep-wake cycle with a resultant increase in stresses, depression and negative emotions [31]. Our observation that students reported changes in sleep pattern and increases in screen use may make the explanations proffered by prior studies applicable to our study findings [31–33]. Studies conducted before the pandemic indicated that changes in sleep pattern increase as age increase [34–36], contradicting the findings of this study. No association between changes in sleep and age was observed.

These findings may have significant implications for the years after the pandemic. Students may have increased risk for multiple cardiometabolic risk and neurocognitive impairment resulting from deterioration of sleep [37, 38]. Increase in screen use may have implications on physical (overweight/obesity, abdominal adiposity, increased body mass index), behavioral (sleeping problems, unhealthy dietary behavior, more sedentary activities and insufficient physical activity), and psychosocial (aggressive behavior, social-emotional delay, hyperactivity-inattention, emotional symptoms, prosocial behavior, peer problems, and conduct problems) health with negative effects [39].

Our findings show that changes in sleep pattern and increases in screen use was significantly higher among people residing in UMICs than in HICs, and lower in LICs/LMICs than HICs. Resources in HICs and UMICs facilitate the use of online programs and platforms such as Zoom, Skype, or Microsoft team for online educational classes and exams and telemedicine to minimize the spread of COVID-19 infection [40]. These online systems were less likely to be implemented in LICs and LMICs due to limited in-country resources and infrastructure [40, 41]. Our findings raise concerns about the possible poor health outcomes resulting from young adults experiencing negative changes in sleep patterns and increases in screen use, especially in UMICs. Of concern is that future adults from UMICs may carry the worst health impact from the pandemic when compared to other economic regions. This is compounded by the fact that UMICs is the only region where respondents reported significantly increased food intake during the pandemic when compared with HICs. Further studies are necessary to better understand the long-term impact of pandemic related behavioural changes among students and the economic regional variations.

The present study also reported a significant increase in sexual activity among students compared to non-students. This is contrary to postulations that fear of contagion will reduce kissing and sexual intercourse between partners [42]. However, sexual activity among young adults, like students, may be driven by desire for intimacy and anxiety [43] and young adults may be more amenable to addressing distance issues by sexting [44] and the use of sex toys [45, 46]. Further studies are needed to understand the reasons for an increase in sexual activity by students during the pandemic and to identify the prevalence of safe sex behaviors among students such as the use of condoms to prevent pregnancy and sexually transmitted infection or the use of pre-exposure prophylaxis to prevent HIV infection. Unwanted teenage pregnancy has increased though it will take time for the epidemiological picture regarding teenage pregnancies to become clear [47]. It is likely that this increase will be highest in LICs/LMICs where access to sexuality education is poor and access to sexual and reproductive health services is limited by sociocultural and political factors [48, 49]. Our study observed higher odds of increased sexual activities in LICs/LMICs when compared with HICs and higher odds of increased sexual activities for females. The increase in females’ sexual activity could be an emotion-driven coping method [25, 50].

Students, older respondents, and those from LICs/LMICs reported lower odds of increased food intake during the pandemic. The reduction in food intake may have resulted from food insecurity due to financial constraints, job loss, salary deductions, restrictions imposed by hiring companies [51, 52], massive rise in the price of food and basic life needs, or illness or death of the main family earner [53]. However, we observed that female respondents had higher odds of increased food intake during the pandemic than non-female respondents contrary to the observation by Flaudias et al [54] who found restrictive eating behaviors among women during the lockdown that might result from loneliness, financial problems, and fear of weight gain. It is also possible that increased food intake may be indicative of mental health challenges [55], a phenomenon that has increased during the pandemic [56] and disproportionately impact females [57].

## Conclusions

The COVID-19 pandemic has resulted in changes in sleep patterns, sexual activity, screen use, and food intake among students worldwide. The pandemic related changes in sleep pattern, screen use, sexual behaviour, and food intake also differed by age, gender and economic regional profile. These findings suggest that the COVID-19 pandemic and associated lockdown restrictions are leading to worse health outcomes for some sub-populations. The health impact may also be amplified for young adults in upper middle-income countries. Further studies would be necessary to fully explain some of the findings presented here, including the paradoxical outcome of age-related associations with increased food consumption during the pandemic. Nevertheless, students have been shown to display an increase in risk of experiencing changes in sleep, sexual behavior, and screen use during the COVID-19 pandemic. This in turn has the potential to lead to wider and more adverse effects on students’ overall wellbeing, thus raising further obligations for higher education provisions.

## Supporting information

S1 Table List of study participants by country and student’s status. (DOCX)

S2 Table Data set of the current study. (XLTX)
