# Supplementary material for: Impact of the COVID-19 pandemic on student’ sleep patterns, sexual activity, screen use, and food intake: A global survey
Source: PLoS One. 2022 Jan 28;17(1):e0262617. doi: 10.1371/journal.pone.0262617 (PMC8797200; doi:10.1371/journal.pone.0262617)
Supplement: S1 Table — (DOCX) [file pone.0262617.s001.docx]

**Supporting Information**

S1 Table. **List of study participants by country and student’s status.**

| **Country classification World Bank 2019** | **Countries** | **Students** | | **Total** |
| --- | --- | --- | --- | --- |
|  |  | No | Yes |  |
| **Low-income countries** | **Afghanistan** | 3 | 1 | 4 |
|  | **Burkina Faso** | 3 | 0 | 3 |
|  | **Congo, Democratic Republic of the** | 1 | 4 | 5 |
|  | **Ethiopia** | 4 | 3 | 7 |
|  | **Gambia** | 9 | 0 | 9 |
|  | **Liberia** | 17 | 1 | 18 |
|  | **Malawi** | 19 | 1 | 20 |
|  | **Mali** | 30 | 2 | 32 |
|  | **Rwanda** | 12 | 3 | 15 |
|  | **Sierra Leone** | 7 | 0 | 7 |
|  | **Sudan** | 186 | 35 | 221 |
|  | **Togo** | 1 | 0 | 1 |
|  | **Uganda** | 63 | 2 | 65 |
|  | **Sub-total** | 355 | 52 | 407 |
|  | | | | |
| **Low- and middle-income countries** | **Algeria** | 6 | 8 | 14 |
|  | **Angola** | 2 | 1 | 3 |
|  | **Bangladesh** | 13 | 5 | 18 |
|  | **Benin** | 12 | 1 | 13 |
|  | **Bhutan** | 0 | 1 | 1 |
|  | **Bolivia** | 3 | 0 | 3 |
|  | **Cambodia** | 5 | 0 | 5 |
|  | **Cameroon** | 11 | 1 | 12 |
|  | **Congo, Republic of the** | 20 | 0 | 20 |
|  | **Cote d'Ivoire** | 32 | 0 | 32 |
|  | **Egypt** | 602 | 158 | 760 |
|  | **Eswatini (formerly Swaziland)** | 4 | 1 | 5 |
|  | **Ghana** | 293 | 71 | 364 |
|  | **Haiti** | 1 | 0 | 1 |
|  | **Honduras** | 1 | 0 | 1 |
|  | **India** | 316 | 449 | 765 |
|  | **Kenya** | 37 | 1 | 38 |
|  | **Laos** | 2 | 0 | 2 |
|  | **Lesotho** | 2 | 0 | 2 |
|  | **Mauritania** | 1 | 0 | 1 |
|  | **Mongolia** | 4 | 1 | 5 |
|  | **Morocco** | 2 | 6 | 8 |
|  | **Myanmar (formerly Burma)** | 5 | 2 | 7 |
|  | **Nepal** | 4 | 0 | 4 |
|  | **Nicaragua** | 1 | 0 | 1 |
|  | **Nigeria** | 4071 | 525 | 4596 |
|  | **Pakistan** | 1128 | 561 | 1689 |
|  | **Philippines** | 247 | 282 | 529 |
|  | **Senegal** | 8 | 2 | 10 |
|  | **Solomon Islands** | 1 | 0 | 1 |
|  | **Sri Lanka** | 1 | 2 | 3 |
|  | **Tanzania** | 20 | 1 | 21 |
|  | **Tunisia** | 2 | 1 | 3 |
|  | **Ukraine** | 10 | 2 | 12 |
|  | **Uzbekistan** | 0 | 1 | 1 |
|  | **Vietnam** | 2 | 4 | 6 |
|  | **Zambia** | 3 | 1 | 4 |
|  | **Zimbabwe** | 40 | 6 | 46 |
|  | **Sub-total** | 6912 | 2094 | 9006 |
|  | | | | |
| **Upper-middle income countries** | **Albania** | 4 | 0 | 4 |
|  | **Argentina** | 479 | 93 | 572 |
|  | **Armenia** | 5 | 0 | 5 |
|  | **Azerbaijan** | 5 | 2 | 7 |
|  | **Belarus** | 14 | 4 | 18 |
|  | **Bosnia and Herzegovina** | 227 | 23 | 250 |
|  | **Botswana** | 27 | 1 | 28 |
|  | **Brazil** | 90 | 20 | 110 |
|  | **Bulgaria** | 2 | 1 | 3 |
|  | **China** | 22 | 13 | 35 |
|  | **Colombia** | 62 | 1 | 63 |
|  | **Dominica** | 1 | 0 | 1 |
|  | **Dominican Republic** | 1 | 0 | 1 |
|  | **Ecuador** | 15 | 0 | 15 |
|  | **Georgia** | 2 | 0 | 2 |
|  | **Indonesia** | 37 | 26 | 63 |
|  | **Iraq** | 9 | 1 | 10 |
|  | **Jordan** | 503 | 210 | 713 |
|  | **Kazakhstan** | 2 | 4 | 6 |
|  | **Kosovo** | 11 | 0 | 11 |
|  | **Lebanon** | 9 | 2 | 11 |
|  | **Libya** | 13 | 1 | 14 |
|  | **Malaysia** | 11 | 6 | 17 |
|  | **Maldives** | 1 | 0 | 1 |
|  | **Mexico** | 456 | 171 | 627 |
|  | **Namibia** | 21 | 1 | 22 |
|  | **North Macedonia**  **(formerly Macedonia)** | 1 | 0 | 1 |
|  | **Paraguay** | 7 | 0 | 7 |
|  | **Peru** | 113 | 9 | 122 |
|  | **Russia** | 1 | 9 | 10 |
|  | **Saint Lucia** | 1 | 0 | 1 |
|  | **Serbia** | 36 | 2 | 38 |
|  | **South Africa** | 409 | 164 | 573 |
|  | **Thailand** | 33 | 4 | 37 |
|  | **Turkey** | 67 | 11 | 78 |
|  | **Sub-total** | 2697 | 779 | 3476 |
|  | | | | |
| **High-income countries** | **Antigua and Barbuda** | 2 | 0 | 2 |
|  | **Australia** | 49 | 5 | 54 |
|  | **Austria** | 7 | 1 | 8 |
|  | **Bahamas** | 0 | 1 | 1 |
|  | **Bahrain** | 12 | 4 | 16 |
|  | **Barbados** | 1 | 0 | 1 |
|  | **Belgium** | 8 | 3 | 11 |
|  | **Canada** | 140 | 19 | 159 |
|  | **Chile** | 145 | 3 | 148 |
|  | **Croatia** | 1 | 0 | 1 |
|  | **Cyprus** | 4 | 1 | 5 |
|  | **Czechia** | 4 | 1 | 5 |
|  | **Denmark** | 5 | 0 | 5 |
|  | **Estonia** | 1 | 1 | 2 |
|  | **Finland** | 305 | 153 | 458 |
|  | **France** | 25 | 6 | 31 |
|  | **Germany** | 39 | 16 | 55 |
|  | **Greece** | 12 | 3 | 15 |
|  | **Hungary** | 79 | 134 | 213 |
|  | **Iceland** | 1 | 2 | 3 |
|  | **Ireland** | 22 | 2 | 24 |
|  | **Israel** | 10 | 4 | 14 |
|  | **Italy** | 13 | 5 | 18 |
|  | **Japan** | 6 | 2 | 8 |
|  | **Lithuania** | 29 | 2 | 31 |
|  | **Luxembourg** | 1 | 0 | 1 |
|  | **Malta** | 0 | 1 | 1 |
|  | **Mauritius** | 6 | 1 | 7 |
|  | **Netherlands** | 36 | 9 | 45 |
|  | **New Zealand** | 16 | 3 | 19 |
|  | **Norway** | 4 | 0 | 4 |
|  | **Oman** | 4 | 1 | 5 |
|  | **Palau** | 1 | 0 | 1 |
|  | **Panama** | 1 | 0 | 1 |
|  | **Poland** | 3 | 2 | 5 |
|  | **Portugal** | 15 | 4 | 19 |
|  | **Qatar** | 25 | 1 | 26 |
|  | **Romania** | 6 | 1 | 7 |
|  | **Saudi Arabia** | 666 | 292 | 958 |
|  | **Singapore** | 1 | 2 | 3 |
|  | **Slovenia** | 3 | 1 | 4 |
|  | **South Korea** | 5 | 1 | 6 |
|  | **Spain** | 18 | 20 | 38 |
|  | **Sweden** | 11 | 5 | 16 |
|  | **Switzerland** | 9 | 0 | 9 |
|  | **Trinidad and Tobago** | 1 | 0 | 1 |
|  | **United Arab Emirates** | 129 | 18 | 147 |
|  | **United Kingdom** | 660 | 92 | 752 |
|  | **United States of America** | 708 | 45 | 753 |
|  | **Uruguay** | 2 | 1 | 3 |
|  | **Sub-total** | 3251 | 868 | 4119 |
|  | **TOTAL** | 13215 | 3793 | 17008 |
